# Sealing the gap: successful transcatheter device closure of baffle leak after ALCAPA repair in an infant—a case report

**DOI:** 10.1093/ehjcr/ytag208

**Published:** 2026-03-30

**Authors:** Samir Bhatia, Philip Roberts, Julian Ayer

**Affiliations:** The Heart Centre for Children, Sydney Children's Hospitals Network, Westmead, NSW 2145, Australia; The Heart Centre for Children, Sydney Children's Hospitals Network, Westmead, NSW 2145, Australia; The Heart Centre for Children, Sydney Children's Hospitals Network, Westmead, NSW 2145, Australia

**Keywords:** Coronary anomaly, Lateral sinus ALCAPA, Takeuchi repair, Baffle leak, Transcatheter closure, Case Report

## Abstract

**Introduction:**

Anomalous origin of the left coronary artery from the pulmonary artery (ALCAPA) is a rare and serious congenital coronary anomaly leading to significant myocardial ischaemia and high mortality if untreated. The Takeuchi repair is an alternative treatment method, although it carries the risk of baffle leaks, a common complication.

**Case summary:**

We present a 7-month-old girl with decompensated heart failure and severe left ventricular (LV) dysfunction, diagnosed with Lateral Sinus ALCAPA. Following the Takeuchi procedure and subsequent extracorporeal membrane oxygenation support, she developed a baffle leak, which was successfully treated using percutaneous transcatheter closure.

**Discussion:**

Baffle leaks are a common complication post-Takeuchi repair of ALCAPA. Early identification and management are crucial. Percutaneous transcatheter closure offers a minimally invasive alternative to surgery for treating baffle leaks. To the best of our knowledge, this is the first documented instance of transcatheter closure for a baffle leak following Takeuchi repair in an infant.

Learning points

- Baffle leak is a known complication after Takeuchi repair for ALCAPA.
- Percutaneous transcatheter closure is a feasible and minimally invasive alternative to surgical reoperation, even in infants.
- This approach may reduce procedural risk and recovery time compared to reoperation.
- The interventional procedure must be performed with great care to avoid coronary artery injury.

## Introduction

Anomalous origin of the left coronary artery (LCA) from the pulmonary artery (ALCAPA) is a serious and rare congenital coronary anomaly that, if not treated, can lead to myocardial ischaemia and increased mortality.^1^ ALCAPA typically presents in infancy with symptoms of heart failure and requires early surgery to prevent long-term cardiac damage.^2^ The standard surgical approach for ALCAPA is the restoration of a two-coronary artery circulation system through direct re-implantation. Takeuchi repair serves as an alternative treatment option in specific cases.^1^ Takeuchi procedure creates an aortopulmonary tunnel to redirect blood flow from the aorta to the anomalous LCA, thereby re-establishing a two-coronary artery system.^3^ The Takeuchi procedure is particularly useful when direct re-implantation is not feasible due to the anatomical position of the coronary arteries. However, it can lead to complications like baffle leaks, which may require additional intervention.^4^Baffle leaks occur in ∼28% of cases post-Takeuchi repair, as mentioned in various studies.^1^ If not quickly addressed, these leaks may cause myocardial ischaemia, heart failure, and pulmonary hypertension.^5^ The early identification and management of such complications are crucial for improving patient outcomes.^6^ This report presents the case of baffle leak in an infant successfully treated with transcatheter device closure, offering a less invasive alternative to surgical reoperation.

## Case presentation

A 7-month-old girl presented to her local hospital with lethargy, increased respiratory effort and poor feeding for last two weeks demonstrating physical signs of heart failure. A transthoracic echocardiogram performed after transfer to our unit revealed anomalous origin of the LCA from the pulmonary artery (ALCAPA) with the LCA arising from the leftward distant pulmonary sinus (*Video 1*; Supplementary material online, *Figure S1*). The left ventricle (LV) was severely dilated with severe systolic dysfunction and moderate mitral regurgitation. (see Supplementary material online, *Video S1*).

After multidisciplinary review, the patient underwent Takeuchi and mitral valve repair via median sternotomy. An intrapulmonary tunnel was created using autologous pericardium to direct flow from the aortopulmonary window to the LCA ostium. Due to persistently poor LV function, the patient was placed on veno-arterial extracorporeal membrane oxygenation which was successfully weaned after 7 days.

**Figure 1:**
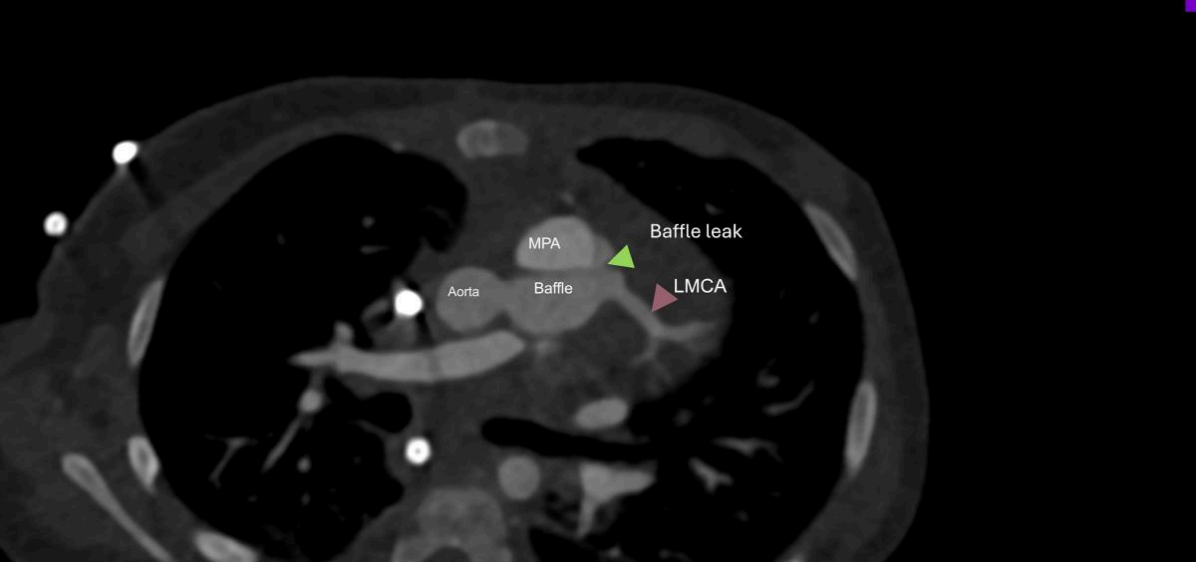
Pre-intervention computed tomography angiography. Post the Takeuchi repair, computed tomography angiography confirms a connection from the baffle into the main pulmonary artery, the diameter of the leak is about 2.2 mm (green arrow). Also seen is the LMCA (pink arrow).

In Immediate post-op period, the child remained clinically unwell with signs of persistent LV dysfunction, inotropic dependence (milrinone) and continued need for non-invasive ventilatory support along with poor weight gain. Clinical examination revealed a gallop rhythm and hepatomegaly, with no audible murmur. Follow-up echocardiography revealed a baffle leak from the intrapulmonary tunnel into the main pulmonary artery, persistent severe LV dysfunction, and moderate mitral regurgitation (see Supplementary material online, *Video S2*). Cardiac magnetic resonance imaging (CMRI) confirmed severely impaired LV function and extensive sub-endocardial late gadolinium enhancement consistent with myocardial scar. The shunt from the baffle leak was hemodynamically significant, with a Qp:Qs ratio of 1.5:1 on CMRI. Computed tomography angiography was undertaken to plan re-intervention, demonstrating a 2.2 mm baffle leak into the main pulmonary artery (*Figure 1*).

Given the recent surgical intervention and elevated risk of reoperation, a percutaneous transcatheter approach was selected to close the baffle leak, 2 months after the initial operation.

**Figure 2:**
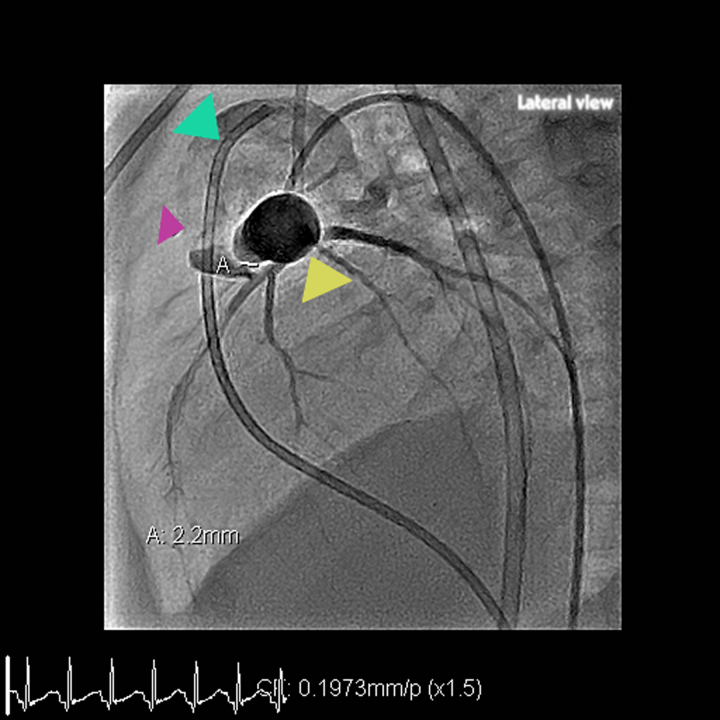
Pre-intervention transcatheter angiography. Percutaneous transcatheter angiography (lateral projection): ascending aortic angiography by cut pigtail (yellow arrow) passed retrogradely via descending aorta through right femoral artery demonstrates contrast leakage from the left coronary artery into the main pulmonary artery and diameter of leak measures 2.2 mm (shown by purple arrow marked as ‘A’). Right coronary artery catheter has been parked in main pulmoanry atery denoted by green arrow.

Under general anaesthesia, 5Fr right femoral artery and 6fr right femoral vein access were obtained. Angiography confirmed the 2.2 mm baffle leak (*Figure 2*, *Video 2*). A 4 Fr Cut pigtail catheter in combination with a Progreat microcatheter was then successfully used to traverse the defect. An arteriovenous loop was established by snaring a Terumo wire in the left pulmonary artery and pulling it back through the leak into the descending aorta. A 4Fr delivery system was negotiated over the wire. A 10 mm snare was utilized on the aortic side to straighten the delivery sheath, allowing for the passage of the device. A 5–2 mm Amplatzer Piccolo™ duct occluder was deployed across the defect under combined fluoroscopic and transoesophageal echocardiographic guidance. Final angiography imaging confirmed complete occlusion of the leak with preserved coronary flow (*Video 3*; Supplementary material online, *Figure S2*).

The patient remained hemodynamically stable throughout the procedure and was returned to the intensive care unit for monitoring. Over the following weeks, she demonstrated steady clinical improvement. Milrinone was successfully weaned, and she was transitioned off non-invasive ventilatory support. She was discharged home one month later on oral captopril, diuretics, and aspirin. At 3-month follow-up, transthoracic echocardiography demonstrated no residual shunt, improvement in mitral regurgitation, and normalization of LV function (see Supplementary material online, *Video S3*).

## Discussion

Anomalous origin of the LCA from the pulmonary artery is a rare congenital heart defect, with an estimated incidence of 1 in every 300 000 live births.^7^ This condition may lead to significant myocardial ischaemia and heart failure if not corrected in early life.^8^ The Takeuchi procedure, which reroutes the anomalous coronary artery to the aorta using a baffle, may be employed where direct anastomosis of the LCA to the aorta is not possible.^9,10^ In our case, Takeuchi repair was selected as the initial surgical procedure after multidisciplinary discussion, as the LCA was found to originate from a distant left pulmonary sinus—identified pre-operatively and confirmed intra-operatively. Ongoing follow-up is essential for patients who have undergone the Takeuchi procedure to monitor for early and late complications such as baffle leaks, coronary artery obstructions, and LV dysfunction.^11^ Following surgery, our patient had persistent severe LV dysfunction requiring inotropic support (milrinone) and non-invasive ventilatory assistance. Serial multimodal imaging—including cardiac CT angiography and cardiac MRI—confirmed a haemodynamically significant baffle leak measuring 2.2 mm, with a Qp:Qs ratio of 1.5:1 into the main pulmonary artery.

The case was re-discussed in multidisciplinary team meetings, and the leak was deemed clinically significant in the context of ongoing LV dysfunction. Given the very short interval (two months) from the initial surgery and the high risks associated with re-sternotomy, consensus was reached to proceed with transcatheter closure, with surgical revision kept as a backup option. The family was informed and consented to the intervention.

Multiple transcatheter occlusion devices are available for the management of residual shunts and baffle leaks. In our case, the Amplatzer Piccolo™ duct occluder was selected because of its low-profile design, compatibility with a 4Fr delivery system, and favourable waist–disc configuration, which allowed secure closure of the baffle leak while minimizing the risk of coronary obstruction or pulmonary valve interference. These features were particularly advantageous in a small infant with limited vascular access. Other options were considered but presented significant limitations: coil embolization carried the risk of incomplete occlusion and embolization, while larger occluder devices required bigger delivery systems, increasing the risk of impingement on adjacent structures.

Our case highlights the importance of multimodal imaging in the detection and quantification of baffle leaks, as well as in planning and guiding interventions. Baffle leaks can result in ongoing myocardial ischaemia and heart failure due to inadequate coronary flow and volume loading of the dysfunctional LV. We present a case of successful management of a Takeuchi baffle leak in an infant using a transcatheter approach. Closure of the baffle leak was temporally associated with clinical improvement in the patient’s condition. To our knowledge, device closure of a baffle leak via a transcatheter approach has only been reported once previously, in an adult following ALCAPA repair.^2^ The transcatheter approach reduces the risks associated with reoperation in the setting of severe LV dysfunction.

In conclusion, our case underscores the complexities of managing ALCAPA and highlights the importance of early detection by multimodality imaging and innovative management strategies for post-surgical complications such as baffle leaks. Transcatheter closure of significant baffle leaks could be considered, even in infants after Takeuchi repair.

## Supplementary Material

ytag208_Supplementary_Data

## Data Availability

The data underlying this article are available in the main manuscript or the Supplementary material online. Due to ethical and privacy concerns, some supporting data cannot be made publicly available but are available from the corresponding authors upon reasonable request.

## Lead author biography

Dr Samir Bhatia is a paediatric cardiologist trained in India with experience working as fellow in Children hospital Westmead Sydney Australia.

## Notes

**Consent:** The patient’s guardian provided written informed consent for the inclusion of the clinical details and photography in this case report in accordance with the Conclusion (COPE) guidelines.
