# Supplementary material for: Sealing the gap: successful transcatheter device closure of baffle leak after ALCAPA repair in an infant—a case report
Source: Eur Heart J Case Rep. 2026 Mar 30;10(4):ytag208. doi: 10.1093/ehjcr/ytag208 (PMC13153465; doi:10.1093/ehjcr/ytag208)
Supplement: ytag208_Supplementary_Data [file ytag208_Supplementary_Data.zip › Supplementary material captions.docx]

Supplementary Figure S1: Transthoracic echocardiography, parasternal short axis view, simultaneous 2D and colour imaging shows anomalous origin of left main coronary artery from lateral pulmonary sinus (Purple arrow)

Supplementary Figure S2: Post device selective coronary angiography of the left main coronary artery in the lateral projection demonstrates no contrast leakage( purple arrow) after implantation of the device (purple star) and device in good position with no obstruction to forward flow.

Supplementary Video S1: Initial transthoracic echocardiogram

Transthoracic echocardiography, apical four chambered view, simultaneous 2D and colour imaging shows dilated LV with severe Dysfunction. Endocardial fibroelastosis of the LV wall. Moderate Mitral regurgitation secondary to annular dilatation

Supplementary Video S2: Post Takeuchi Repair

Transthoracic echocardiography, High parasternal short axis view, colour imaging shows leak from the baffle into the main pulmonary artery.

Supplementary Video S3: Follow up transthoracic echocardiogram at 1 year

Transthoracic echocardiography, apical four chambered view, simultaneous 2D and colour imaging shows normal LV function with trivial Mitral regurgitation
