# Supplementary figures and images for: Sealing the gap: successful transcatheter device closure of baffle leak after ALCAPA repair in an infant—a case report
Source: Eur Heart J Case Rep. 2026 Mar 30;10(4):ytag208. doi: 10.1093/ehjcr/ytag208 (PMC13153465; doi:10.1093/ehjcr/ytag208)

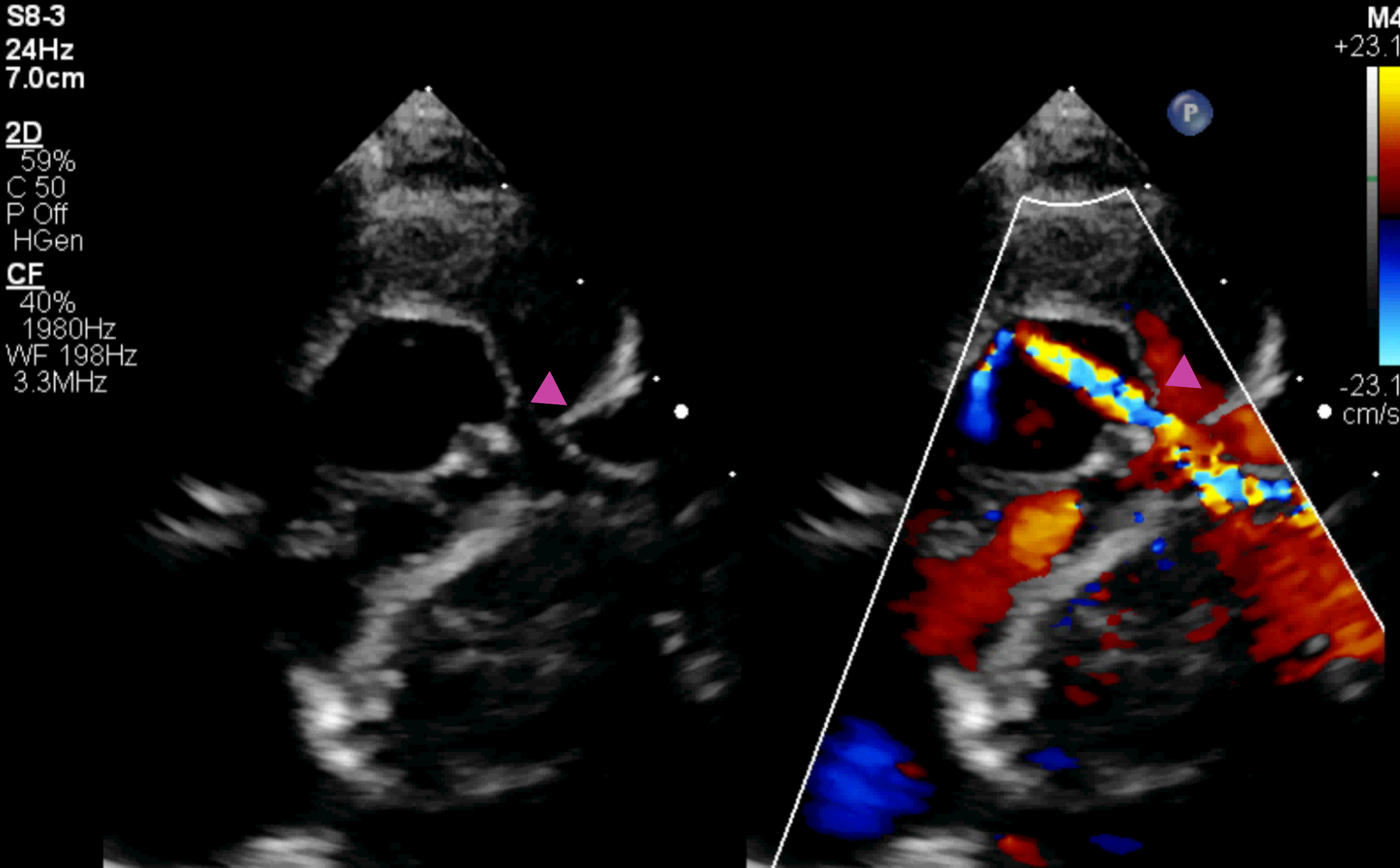

Supplement: ytag208_Supplementary_Data [file ytag208_Supplementary_Data.zip › figure S1.png]

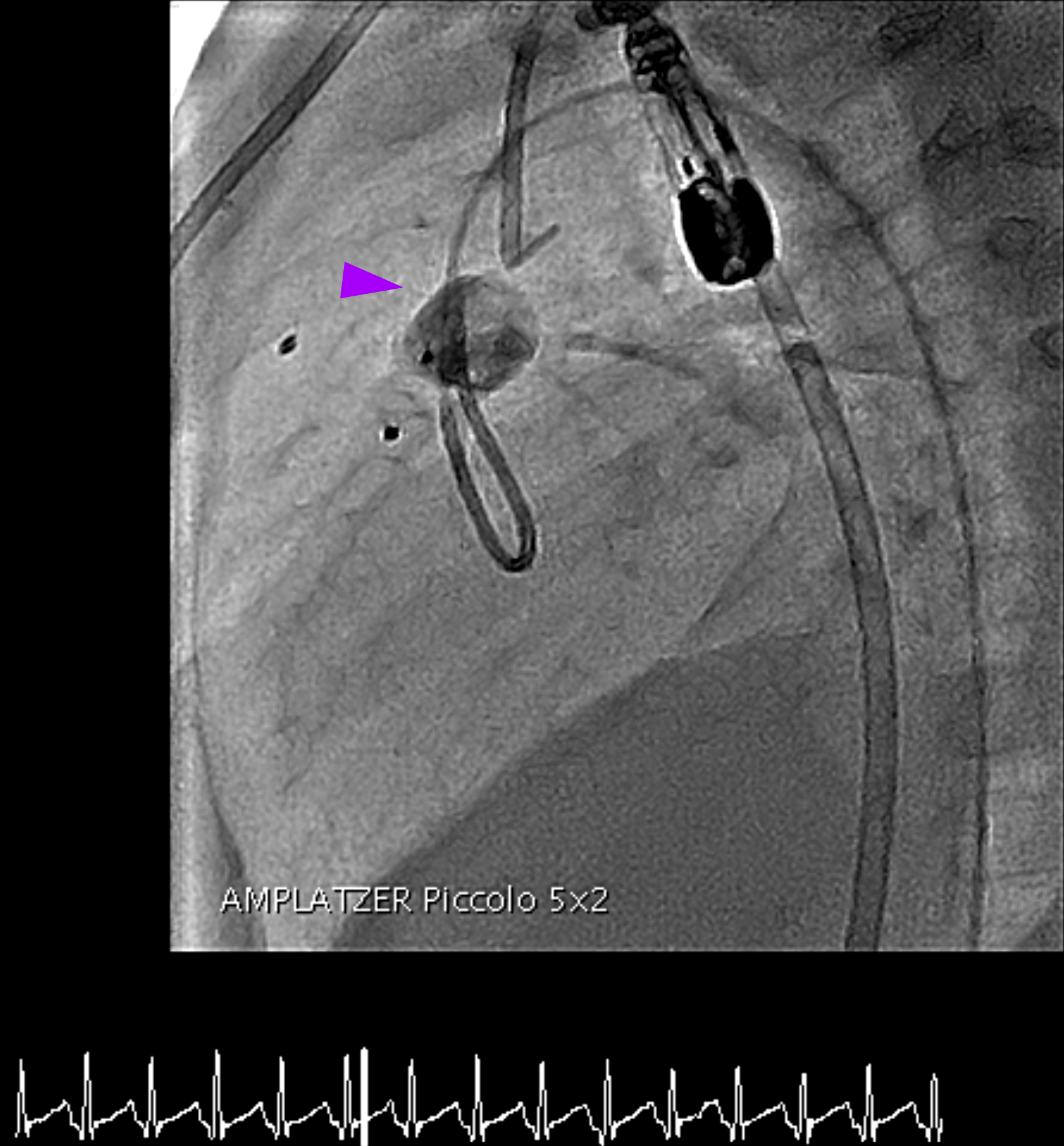

Supplement: ytag208_Supplementary_Data [file ytag208_Supplementary_Data.zip › Figure S2.png]
